# Factors associated with hypothermia within the first 6 hours of life in infants born at ≥34^0^ weeks’ gestation: a multivariable analysis

**DOI:** 10.1186/s12887-022-03512-x

**Published:** 2022-07-25

**Authors:** Laura Nguyen, Nicholas Mitsakakis, Ewa Sucha, Brigitte Lemyre, Sarah Linda Lawrence

**Affiliations:** 1 Faculty of Medicine, University of Ottawa, 451 Smyth Rd, Ottawa, Ontario Canada; 2 Children’s Hospital of Eastern Ontario Research Institute, 401 Smyth Rd, Ottawa, Ontario Canada; 3 The Ottawa Hospital, General Campus, 501 Smyth Road, Ottawa, Ontario Canada; 4 Department of Pediatrics, Children’s Hospital of Eastern Ontario, 401 Smyth Rd, Ottawa, Ontario, K1H 8L1 Canada

**Keywords:** Neonatal, Newborn, Hypothermia, Risk factors, Morbidity, Multivariable analysis

## Abstract

**Background:**

Lack of appropriate temperature management has been associated with significant adverse outcomes in preterm and low birthweight neonates. There is a lack of similar investigations in the late preterm (34^0^–36^6^) and term (≥37^0^) neonate population. Our aim was to identify key risk factors as well as clinical outcomes associated with hypothermia in this population.

**Methods:**

A retrospective chart review was conducted at the Ottawa Hospital including all eligible infants ≥34^0^ weeks’ gestation over a one-month period in November 2020. Infant, maternal, and delivery room variables were collected, including prematurity, maternal temperature, delivery mode, birthweight, and premature rupture of membranes, as well as clinical outcomes such as NICU/SCN admission and length of stay. Regression models were generated, adjusted for covariates, and stepwise regression was performed.

**Results:**

Four hundred forty infants were included in the analysis; 26.8% (118/440) were hypothermic within 6 hours of delivery. In the multivariable analysis, prematurity, low 5 minute Apgar score (< 7) or need for resuscitation, maternal hypertension, and absence of premature rupture of membranes > 18 hours or suspected maternal infection were significantly associated with hypothermia within 6 hours of delivery (*p* < 0.05). Multivariable analysis of clinical outcomes demonstrated a significant association between hypothermia within 6 hours of delivery and NICU/SCN admission (OR = 2.87; 95% CI 1.36, 6.04), need for respiratory support or diagnosis of respiratory distress syndrome (OR = 3.94; 95% CI 1.55, 10.50), and length of stay (exp(β) = 1.20; 95% bootstrap CI 1.04, 1.37).

**Conclusions:**

Our results suggest there are similar factors associated with hypothermia in our study population of infants born at ≥34^0^ weeks’ gestation compared to prior studies in preterm and low-birthweight infants. Furthermore, hypothermia is associated with higher risk of adverse outcomes, which highlights the need to prevent hypothermia in all newborns.

**Supplementary Information:**

The online version contains supplementary material available at 10.1186/s12887-022-03512-x.

## Background

Neonatal hypothermia is a common problem associated with several short- and long-term clinical outcomes [1–3]. The World Health Organization defines neonatal hypothermia as an axillary temperature below 36.5 °C among newborns aged below 28 days [4]. Studies have shown a direct relationship between the degree of hypothermia and the risk of infant mortality [2, 5, 6]. Every 1° drop in body temperature below 36.5 °C has been found to increase the risk of mortality in low birthweight infants by 28% [2]. Other clinical outcomes associated with neonatal hypothermia include late-onset sepsis, respiratory disease, hypoglycemia, and intraventricular hemorrhage [7, 8].

Past studies have suggested a number of risk factors associated with hypothermia in neonates, including maternal hypertension, antenatal use of steroids, low maternal temperature, inadequate antenatal care, low birth weight, prematurity, need for resuscitation, nighttime delivery, delivery during cold season, low birth weight, and prematurity [1, 6, 9–14]. Warmer delivery room or resuscitation room temperatures and warm transport to NICU have been found to be protective against neonatal hypothermia [12, 15, 16].

Rates of hypothermia upon admission to Neonatal Intensive Care Units (NICUs) are particularly prevalent in low birthweight infants and can reach up to 50%, as demonstrated by multiple large multi-center studies in developed countries [1, 2, 17]. The Ottawa Hospital (TOH) is an academic institution with a tertiary-care perinatal unit with a level 3 NICU, as well as a level 2 Special Care Nursery (SCN) housed in two campuses, with over 6000 deliveries annually. Over the past 3 years, the rate of hypothermia upon NICU/SCN admission at these two centres in the > 34^0^ weeks’ gestation population has been approximately 40%. Many prior studies have focused on high-risk populations, such as low-birthweight or preterm infants, however there is a lack of investigations focusing on hypothermia in the late preterm (34^0^–36^6^), early term (37^0^–38^6^), and full term (39^0^+) neonate population. Our objective was to therefore identify protective and risk factors associated with hypothermia in infants born at **≥**34^0^ weeks’ gestation, as well as determine whether hypothermia within 6 hours of life is associated with poor clinical outcomes in this population.

## Methods

### Study design

We conducted a retrospective chart review that included all infants delivered between November 1 and November 30, 2020, at TOH Civic (TOHCC) and General Campuses (TOHGC) who were 34^0^ weeks’ gestation or above. Infants with birth defects involving open wall defects (i.e., gastroschisis, neural tube defects), where palliative care was chosen from delivery, or who were diagnosed with any degree of hypoxic ischemic encephalopathy, using Sarnat staging criteria, were excluded. Data was collected on de-identified case study forms created on REDCap (a secure, web-based application for building and storing data).

### Data

Data collected through patient charts on EPIC electronic medical records system included demographic characteristics and the following infant, maternal, and intrapartum/delivery room variables: prematurity (defined as less than 37^0^ weeks’ gestation), low maternal temperature (< 36.5 °C) within 1 hour of delivery, delivery mode (planned c-section, emergency c-section, or vaginal delivery), birthweight (in grams), intrauterine growth restriction (IUGR) status, low Apgar at 5 min (score < 7) or need for resuscitation (requiring any of the following prior to transfer to ward/NICU: supplementary O_2_, CPAP, PPV, intubation, chest compressions, epinephrine), gestational diabetes, maternal hypertension, delayed cord clamping (> 30 seconds), early skin-to-skin (any duration of contact prior to transfer to ward/NICU), premature rupture of membranes (PROM) > 18 hours or suspected maternal/uterine infection (any documentation of chorioamnionitis in the mother’s chart; maternal axillary temp ≥38.0 °C, with fetal or maternal tachycardia, and leukocytosis; uterine tenderness; or documentation of foul smelling amniotic fluid), cord arterial pH ≤7.15 or base deficit ≥10, and maternal exposure to epidural anesthetic. The main outcome was hypothermia (< 36.5 °C) within 6 hours of delivery. At TOHGC and TOHCC, neonatal axillary temperatures are routinely measured, using either the Filac or Welch-Allyn portable thermometers. Clinical outcomes included: low blood glucose (≤2.6 mmol/L) within the first 2 and 12 hours, NICU/SCN Admission, need for respiratory support (after transfer to ward or NICU) or diagnosis of respiratory distress syndrome (RDS) (supported by clinical and x-ray findings), and hospital length of stay. Observations with missing values in outcome or any of the covariates were excluded from the analysis.

### Sample size

Following the rule of a ratio requiring at least 15 between the “limiting sample size” (the size of the smallest class between those with and without hypothermia) and the number of predictors, and assuming that the prevalence of those with hypothermia in the TOHGC/TOHCC population is around 40% based on estimates from the Better Outcomes Registry and Network (BORN) database, a sample size of 400 patients (corresponding to about 160 patients with hypothermia) was calculated to allow for the reliable investigation of 13 predictors.

### Statistical methods

The association between hypothermia and each variable of interest was assessed using appropriate statistical tests (i.e., Chi-square test for categorical outcomes and Student’s T-test or Wilcoxon test for continuous outcomes). Proper regression models were generated, adjusted for additional covariates with previously known association with the outcomes. Skewed continuous outcomes (e.g., time) were transformed appropriately. Subsequently, a stepwise regression was performed, beginning with a full model including all factors. The factors in the final model were selected using Akaike information criterion (AIC) and a combination of the forward and backward variables selection approach. Subgroup analysis was performed for late preterm infants (34^0^–36^6^ weeks’ gestation), and term infants (≥37^0^ weeks’ gestation). Due to the low number of late preterm infants included in our study, only univariate analysis was carried out for the subgroups.

## Results

A total of 455 infants were delivered at TOHGC and TOHCC in November 2020; 440 infants delivered at greater than or equal to 34^0^ weeks’ gestation at TOHGC (*n* = 188) and TOHCC (*n* = 252) were included in our analyses. Characteristics of the included infants are presented in Table 1. The final cohort had a mean (standard deviation (SD)) birthweight and gestational age of 3368.7 g (529.0) and 39.3 weeks (1.5) respectively. The mean (SD) lowest temperature within 6 hours for all infants was 36.6 °C (0.3 °C), ranging from 35.5 °C to 37.6 °C. 72.7% (320/440) of the study population had a temperature in the World Health Organization recommended range of 36.5 °C to 37.5 °C. A total of 26.8% (118/440) of infants were hypothermic within 6 hours of delivery. One infant was hyperthermic within 6 hours of delivery (> 37.5 °C). A total of 49 infants were admitted to the NICU/SCN and of these, 22.4% (11/49) were hypothermic at time of admission. Of the infants admitted to the NICU/SCN, the mean (SD) admission temperature was 36.8 °C (0.6 °C). In the subgroup of late preterm infants (*n* = 34), 62% (21/34) of infants were hypothermic within 6 hours of delivery, compared to 24% (97/406) of term infants.

**Table 1:** Demographic and clinical characteristics of included infants

Characteristic	Included Infants (***n*** = 440)	Subgroup of Late Preterm Infants (34^**0**^–36^**6**^ weeks’ gestation) (***n*** = 34)
Maternal Age in years, mean (SD)	32.2 (5.3)	31.9 (6.6)
Birthweight in grams, mean (SD)	3368.7 (529.0)	2462.7 (473.6)
Gestational Age in weeks, mean (SD)	39.3 (1.5)	36.0 (0.8)
Time of Lowest Temperature Measured in minutes, mean (SD)	129.29 (96.8)	186.8 (200.1)
Infant Sex		
Male, n (%)	226 (51.3)	20 (58.8)
Female, n (%)	214 (48.7)	14 (41.2)
Multiple Birth, n (%)	24 (5.5)	14 (41.2)
Mode of Delivery		
Vaginal delivery, n (%)	299 (67.8)	14 (41.2)
Planned C-section, n (%)	69 (15.8)	12 (35.3)
Emergency C-section, n (%)	72 (16.4)	8 (23.5)
Cord Arterial pH, mean (SD)	7.24 (0.07)	7.26 (0.05)
Base Deficit in mmol/L, mean (SD)	3.41 (2.99)	1.83 (2.76)
Hospital Length of Stay in hours, mean (SD)	40.4 (31.4)	87.8 (77.5)
Length of Ventilation in hours, mean (SD)	0.6 (5.4)	4.0 (16.7)

Preliminary univariate analysis demonstrated a significant association (*p* < 0.05) between increased risk of hypothermia within the first 6 hours and prematurity, low maternal temperature, mode of delivery, low birth weight, 5 minute Apgar score less than 7, maternal hypertension, and need for resuscitation (see Table 2). Early skin-to-skin contact and PROM > 18 hours was associated with lower risk of hypothermia (see Table 2). In univariate analysis of late preterm infants, only low birth weight was significantly associated to hypothermia within the first 6 hours (*p* = 0.015) (see Table 1 in Additional File 1). In univariate analysis of term infants, maternal hypertension, mode of delivery, need for resuscitation, low birth weight was significantly associated to increased risk of hypothermia within the first 6 hours (*p* < 0.05); early skin-to-skin was associated with lower risk of hypothermia (*p* = 0.05) (see Table 2 in Additional File 1).

**Table 2:** Univariate analysis of infant/maternal/intrapartum variables

Infant/Maternal/Intrapartum Variables	Infants who had hypothermia (***n*** = 118)	Infants who did not have hypothermia (***n*** = 321)	***p***-value
Prematurity, n (%)	21 (17.8)	13 (4.0)	< 0.001*
Low Maternal Temperature Within 1 Hour of OR, n (%)^a^	5 (4.2)	7 (2.2)	0.044*
Mode of Delivery			0.001*
Vaginal Delivery, n (%)	64 (54.2)	234 (72.9)	
Planned C-Section, n (%)	28 (23.7)	44 (13.7)	
Emergency C-Section, n (%)	26 (22.0)	43 (13.4)	
Birth Weight (in grams), mean (SD)	3139.8 (621.1)	3452.7 (466.2)	< 0.001*
IUGR Status, n (%)	10 (8.5)	12 (3.7)	0.051
Low 5-Minute Apgar, n (%)	7 (5.9)	3 (0.9)	0.005*
Gestational Diabetes, n (%)	14 (11.9)	30 (9.3)	0.474
Maternal Hypertension, n (%)	25 (21.2)	29 (9.0)	< 0.001*
Delayed Cord Clamping, n (%)^b^	107 (90.7)	290 (91.2)	0.852
Need for Resuscitation, n (%)	23 (19.5)	22 (6.9)	< 0.001*
Early Skin-to-Skin Contact, n (%)^c^	90 (76.3)	287 (90.0)	< 0.001*
PROM > 18 Hours, n (%)	8 (6.8)	45 (14.0)	0.047*
Suspected Maternal or Uterine Infection, n (%)	5 (4.2)	13 (4.0)	1
Cord Arterial pH, mean (SD)	7.24 (0.08)	7.24 (0.07)	0.715
Arterial Cord Base Deficit in mmol/L, mean (SD)	2.84 (2.96)	3.62 (2.98)	0.007*
Epidural Anesthetic, n (%)	104 (88.1)	287 (89.4)	0.731

Note: ^a^Missing values for 368 patients, ^b^Missing values for 3 patients, ^c^Missing values for 2 patients, *p-value <0.05

For clinical outcomes, univariate analysis demonstrated a significant association (*p* < 0.05) between hypothermia within the first 6 hours and risk of hypoglycemia within the first 2 and 12 hours, need for IV therapy for hypoglycemia, increased length of stay, neonatal intensive care unit/special care nursery admission, need for respiratory support, length of non-invasive ventilation, and diagnosis of RDS (see Table 3). In univariate analysis of late preterm infants, hypothermia was not significantly associated with any clinical outcomes (see Table 3 in Additional File 1). In term infants, hypothermia within the first 6 hours was significantly associated with need for IV therapy for hypoglycemia and NICU/SCN admission (see Table 4 in Additional File 1).

**Table 3:** Univariate analysis of clinical outcomes

Clinical Outcomes	Infants who had hypothermia (***n*** = 118)	Infants who did not have hypothermia (***n*** = 321)	***p***-value
Need for IV Therapy for Hypoglycemia, n (%)	6 (5.1)	3 (0.9)	0.014*
NICU/SCN Admission, n (%)	26 (22.0)	23 (7.2)	< 0.001*
Length of Stay in hours, mean (SD)	36.5 (21.8)	51.1 (47.4)	< 0.001*
Need for Respiratory Support, n (%)	11 (9.3)	7 (2.2)	0.002*
Transfer to Another Hospital, n (%)	5 (4.2)	7 (2.2)	0.319
Non-Invasive Ventilation Duration in hours, mean (SD)	0.2 (1.5)	1.9 (10.2)	< 0.001*
Metabolic Acidemia, n (%)	2 (1.7)	4 (1.2)	0.662
Diagnosis of RDS, n (%)	4 (3.4)	1 (0.3)	0.02*
Hypoglycemia ≤2.6 mmol/L in First 2 Hours, n (%)	14 (11.9)	9 (2.8)	< 0.001*
Hypoglycemia ≤2.6 mmol/L in First 12 Hours, n (%)	18 (15.3)	19 (5.9)	0.003*

Note: *p-value <0.05

In the multivariable analysis, prematurity, low 5 minute Apgar or need for resuscitation, and maternal hypertension remained significantly associated with hypothermia within 6 hours of delivery; PROM > 18 hours or suspected maternal infection was significantly associated with lower risk of hypothermia within 6 hours of delivery (see Table 4). The adjusted ORs after AIC-based stepwise regression of prematurity and low 5 minute Apgar or need for resuscitation increased to 4.54 (95% CI 2.10–10.2, *p* < 0.001) and 2.91 (1.41–5.98, *p* = 0.003) for infants with hypothermia within 6 hours of delivery, respectively.

**Table 4:** Multivariable analysis of infant/maternal/intrapartum variables

Characteristic	OR^**a**^	95% CI^**b**^	***p***-value
Prematurity	4.42	1.87, 10.8	< 0.001*
Mode of Delivery			
Vaginal Delivery (reference)	–	–	–
Planned C-Section	1.85	0.91, 3.71	0.085
Emergency C-Section	1.91	0.93, 3.87	0.073
IUGR Status	1.71	0.59, 4.78	0.3
Low 5 Minute Apgar OR Need for Resuscitation	2.55	1.08, 6.09	0.033*
Gestational Diabetes	0.87	0.37, 1.92	0.7
Delayed Cord Clamping	1.27	0.53, 3.34	0.6
Early Skin-to-Skin	1.30	0.58, 3.03	0.5
PROM > 18 Hours OR Suspected Maternal/Uterine Infection	0.40	0.17, 0.86	0.027*
Cord Arterial pH ≤ 7.15 OR Arterial Cord Base Deficit ≥10 mmol/L	1.34	0.57, 3.01	0.5
Epidural Anesthetic	0.60	0.26, 1.45	0.2
Maternal Hypertension	2.40	1.21, 4.72	0.011*

Note: ^a^OR = Odds Ratio; ^b^CI = Confidence Interval; *p-value <0.05

Multivariable analysis of clinical outcomes demonstrated significant association of NICU/SCN admission and need for respiratory support or diagnosis of RDS. These analyses were controlled for covariates (see Table 5). A log transformed response model demonstrated significant association between hypothermia within 6 hours of delivery and hospital length of stay (exp(β) = 1.20, 95% bootstrap CI 1.04–1.37, *p* = 0.021), with adjustment for gestational age, birthweight, and infant sex.

**Table 5:** Multivariable analysis of clinical outcomes

Characteristic	Risk Statistic	95% CI^f^	***p***-value
Low Glucose (≤2.6 mmol/L) Within First 12 Hours^a^	OR^e^ = 2.07	0.96, 4.40	0.059
NICU/SCN Admission^b^	OR^e^ = 2.87	1.36, 6.04	0.006*
Need for Respiratory Support or Diagnosis of RDS^c^	OR^e^ = 3.94	1.55, 10.50	0.004*
Length of Stay^d^	exp(β) = 1.20	1.04, 1.37	0.021*

Note: ^a^controlled for maternal gestational diabetes and birth weight; ^b^excluding automatic NICU/SCN admissions (weight < 2.2 kg or ≤ 35^6^ weeks’ gestation); ^c^controlled for maternal gestational diabetes; ^d^controlled for gestational age, birthweight, infant sex; ^e^OR = Odds Ratio; ^f^CI = Confidence Interval; ^*^p-value <0.05

## Discussion

To the best of our knowledge, this is the first study to investigate the association between infant, maternal, and intrapartum/delivery room variables, and hypothermia in infants delivered at greater than or equal to 34^0^ weeks’ gestation in North America. In our cohort, the rate of hypothermia was 26.8%, which was lower than the predicted rate of 40%, however, still involved a high proportion of infants. Of note, there was a difference between the two campuses, with 38.3% (72/188) of infants from TOHGC and 23.0% (58/252) from TOHCC being hypothermic within 6 hours of delivery. As there is a difference between the two campuses, further quality improvement studies to investigate strategies to standardize and improve neonatal thermoregulation at these two centers would be of merit. A larger proportion of late preterm infants were hypothermic (62%) compared with term infants (24%).

Our study found similar risk factors for neonatal hypothermia compared to prior studies in preterm and low birthweight infants, including prematurity, suspected maternal infection, and maternal hypertension. A combination of factors contributes to the rapid fall in body temperature in newborns, including the evaporation of fluid from the infant’s wet skin, convective heat loss to the relatively cold air in the delivery or operating room, radiation from nearby cool surfaces, and conductive heat loss from cold surfaces in contact with the infant [18]. Newborn infants have a relatively large surface area to body mass ratio and thin, immature skin, as well as decreased subcutaneous fat layers, predisposing them to rapid heat loss [4]. Past research has suggested that premature infants have thinner, more immature skin as well as decreased ability for temperature control by shivering, and lower glycogen stores and adipose tissue [19]. Similarly, the need for resuscitation has been associated with higher rates of hypothermia in very low birth weight infants ≥26^0^ weeks’ gestation (*p* = 0.02) [13]. It has been suggested that infants who receive more intervention in the delivery room (e.g. intubation, chest compressions, medications) could be at risk of hypothermia due to less attention to temperature control during resuscitation, as well as exposure to cool, non-humidified gases when ventilation is performed in the delivery room [20]. In addition, asphyxia can decrease oxygen concentration which decreases metabolic rate and can lead to heat loss [21]. On the other hand, intrapartum infection (chorioamnionitis) has been found to be protective against hypothermia in neonates which is hypothesized to be due to resulting inflammatory responses, leading to higher core maternal and neonatal temperatures during delivery and at birth [13]. Due to the low number of late preterm infants included in our study (*n* = 34), it is difficult to draw conclusions from the subgroup analysis. It is interesting to note that in the subgroup of term infants, early skin-to-skin was associated with lower risk of hypothermia. This is in keeping with previous studies suggesting improvements in thermal control and physiological stability in healthy term or stabilized preterm infants, who received immediate, uninterrupted skin-to-skin contact after birth [7].

In our results, while birth weight and IUGR status was associated with higher risk of hypothermia within 6 hours of delivery, this relationship did not reach statistical significance in the multivariable analysis. Previous investigations have suggested that infants with small birth weight have large surface area to body mass ratio which makes them prone to rapid heat loss and hypothermia [22]. It has also been suggested that low birth weight babies have decreased thermal insulation due to less subcutaneous fat and reduced amount of brown fat [22]. However, our results are in line with a prior study by Merazzi et al. in healthy term or late preterm infants (≥ 35^0^ weeks’ gestation), which found that low birth weight (< 2500 g) was not significantly associated with neonatal hypothermia at nursery entry (OR = 1.25 95% CI: 0.85–1.83, *p* = 0.26) [16]. These findings could suggest that birth weight has smaller effect on temperature regulation in healthy term and late preterm infants.

Our data also found associations between several clinical outcomes and hypothermia within 6 hours of delivery, including higher risk of NICU/SCN admission, need for respiratory support or RDS, and longer hospital length of stay. In our study, neonates with hypothermia tended to stay approximately 20% longer in hospital than neonates without hypothermia. This is in keeping with prior studies which have found that neonatal hypothermia is associated with poor clinical outcomes, including increased risk of late onset sepsis, respiratory disease, hypoglycemia, and intraventricular hemorrhage [7, 8]. Notably, longer postpartum hospital length of stay and NICU admissions have implications for cost and resource utilization as they have been associated with higher cost of hospitalization in several studies previously conducted in Canada [23, 24].

The strengths of our study include inclusion of data from multiple centers and inclusion of late preterm and term babies, which have not been the focus in previous investigations on neonatal hypothermia even though they represent a majority of infants delivered and admitted to NICU in Canada [25]. Our results can be relevant to similar tertiary centres in North America. As well, we collected data for a broad range of risk factors and clinical outcomes, allowing us to control for relevant covariates in our multivariable analysis. Some limitations include the study’s retrospective nature, and omission from our analysis of some variables found to have a significant association with neonatal hypothermia in prior studies, due to missing data or low number of outcomes. These include low maternal temperature within 1 hour of delivery and infant mortality. This may be because our cohort did not include infants < 34^0^ weeks’ gestation compared to prior studies in high-risk infants. As well, timing of temperature measurement was not predefined, except upon admission to NICU if applicable. We chose a period of 6 hours after delivery to attempt to capture a relevant time period following delivery that would be most affected by the delivery room or intrapartum variables collected in our study; this period has also been used in prior studies [11, 15]. Since we chose to investigate hypothermia within the first 6 hours of life, caution needs to be taken when drawing comparisons to prior studies which have investigated hypothermia upon NICU/SCN admission as there may be other factors affecting temperatures upon NICU/SCN admission that were not captured within our data. In addition, some variability in temperatures between infants may be due to type of thermometer used [26, 27], which was not reliably documented in our patient charts and therefore not controlled in our data. Additionally, at our hospitals, we do not have heated gases in the delivery room, and at the time of the study, we had no documentation of room temperatures for the delivery room, NICU/SCN, or well-infant nursery. These two variables have been suggested to affect temperature control and may be a confounding factor [28]. In the previous year, we have started collecting this data and have noted average temperatures of 20–21 °C in the birthing unit operating room and 23–24 °C in the neonatal resuscitation room.

## Conclusion

We found that infants 34^0^ weeks’ gestation have similar risk factors for hypothermia shortly after birth compared with those of smaller gestational age, including prematurity, maternal hypertension, and low Apgar score or need for resuscitation. Because neonatal hypothermia is associated with an increased risk of several adverse clinical outcomes such as NICU/SCN admission, increased length of stay, hypoglycemia and respiratory distress, providers should continue to consider hypothermia a risk factor that should be addressed during the immediate postnatal period in infants of all gestational ages. Future research should seek to identify quality improvement strategies to minimize risk factors or reduce hypothermia in high-risk infants in order to improve clinical outcomes in neonates.

## Supplementary Information

**Additional file 1.**

## Data Availability

The datasets used and/or analyzed during the current study are available from the corresponding author on reasonable request.

## Abbreviations

NICU: Neonatal Intensive Care Unit
SCN: Special Care Nursery
TOHCC: The Ottawa Hospital Civic Campus
TOHGC: The Ottawa Hospital General Campus
IUGR: intrauterine growth restriction
PROM: premature rupture of membranes
RDS: respiratory distress syndrome
AIC: Akaike information criterion
OR: odds ratio
CI: confidence interval
SD: standard deviation
