# Supplementary material for: Factors associated with hypothermia within the first 6 hours of life in infants born at ≥340 weeks’ gestation: a multivariable analysis
Source: BMC Pediatr. 2022 Jul 25;22:447. doi: 10.1186/s12887-022-03512-x (PMC9316355; doi:10.1186/s12887-022-03512-x)
Supplement: Supplementary file 1 — Additional file 1. [file 12887_2022_3512_MOESM1_ESM.docx]

**Table 1:** Univariate Analysis of Infant/Maternal/Intrapartum Variables in Late Preterm Infants

| **Infant/Maternal/Intrapartum Variables** | **Infants who had hypothermia (n=118)** | **Infants who did not have hypothermia (n=321)** | **p-value** |
| --- | --- | --- | --- |
| Low Maternal Temperature Within 1 Hour of OR, n (%)^1^ | 1 (4.8) | 0 (0) | 1 |
| Mode of Delivery |  |  | 0.142 |
| Vaginal Delivery, n (%) | 7 (33.3) | 7 (53.8) |  |
| Planned C-Section, n (%) | 10 (47.6) | 2 (15.4) |  |
| Emergency C-Section, n (%) | 4 (19.0) | 4 (30.8) |  |
| Birth Weight (in grams), mean (SD) | 2318.0 (417.1) | 2696.3 (480.6) | 0.015* |
| IUGR Status, n (%) | 5 (23.8) | 1 (7.7) | 0.37 |
| Low 5-Minute Apgar, n (%) | 3 (14.3) | 0 (0) | 0.27 |
| Gestational Diabetes, n (%) | 4 (19.0) | 1 (7.7) | 0.627 |
| Maternal Hypertension, n (%) | 8 (38.1) | 3 (23.1) | 0.465 |
| Delayed Cord Clamping, n (%) | 20 (95.2) | 13 (100.0) | 1 |
| Need for Resuscitation, n (%) | 6 (28.6) | 1 (7.7) | 0.21 |
| Early Skin-to-Skin Contact, n (%) | 8 (38.1) | 6 (46.2) | 0.728 |
| PROM >18 Hours, n (%) | 1 (4.8) | 3 (23.1) | 0.274 |
| Suspected Maternal or Uterine Infection, n (%) | 0 (0) | 1 (7.7) | 0.382 |
| Cord Arterial pH, mean (SD) | 7.3 (0.1) | 7.3 (0.0) | 0.624 |
| Arterial Cord Base Deficit in mmol/L, mean (SD) | 1.6 (2.3) | 2.3 (3.4) | 0.254 |
| Epidural Anesthetic, n (%) | 20 (95.2) | 13 (100.0) | 1 |

Note: ^1^Missing values for 31 patients, *p-value ≤ 0.05

**Table 2:** Univariate Analysis of Infant/Maternal/Intrapartum Variables in Term Infants

| **Infant/Maternal/Intrapartum Variables** | **Infants who had hypothermia (n=118)** | **Infants who did not have hypothermia (n=321)** | **p-value** |
| --- | --- | --- | --- |
| Low Maternal Temperature Within 1 Hour of OR, n (%)^1^ | 4 (4.1) | 7 (2.3) | 0.102 |
| Mode of Delivery |  |  | 0.016* |
| Vaginal Delivery, n (%) | 57 (58.8) | 227 (73.7) |  |
| Planned C-Section, n (%) | 18 (18.6) | 42 (13.6) |  |
| Emergency C-Section, n (%) | 22 (22.7) | 39 (12.7) |  |
| Birth Weight (in grams), mean (SD) | 3317.8 (501.2) | 3484.6 (438.5) | 0.002* |
| IUGR Status, n (%) | 5 (5.2) | 11 (3.6) | 0.55 |
| Low 5-Minute Apgar, n (%) | 4 (4.1) | 3 (1.0) | 0.06 |
| Gestational Diabetes, n (%) | 10 (10.3) | 29 (9.4) | 0.844 |
| Maternal Hypertension, n (%) | 17 (17.5) | 26 (8.4) | 0.022* |
| Delayed Cord Clamping, n (%)^2^ | 87 (89.7) | 277 (90.8) | 0.695 |
| Need for Resuscitation, n (%) | 17 (17.5) | 21 (6.8) | 0.004* |
| Early Skin-to-Skin Contact, n (%)^3^ | 82 (84.5) | 281 (91.8) | 0.05* |
| PROM >18 Hours, n (%) | 7 (7.2) | 42 (13.6) | 0.108 |
| Suspected Maternal or Uterine Infection, n (%) | 5 (5.2) | 12 (3.9) | 0.568 |
| Cord Arterial pH, mean (SD) | 7.2 (0.1) | 7.2 (0.1) | 0.854 |
| Arterial Cord Base Deficit in mmol/L, mean (SD) | 3.2 (3.0) | 3.7 (3.0) | 0.091 |
| Epidural Anesthetic, n (%) | 84 (86.6) | 274 (89.0) | 0.585 |

Note: ^1^Missing values for 368 patients, ^2^Missing values for 3 patients, ^3^Missing values for 2 patients, *p-value ≤ 0.05

**Table 3:** Univariate Analysis of Clinical Outcomes in Late Preterm Infants

| **Clinical Outcomes** | **Infants who had hypothermia (n=118)** | **Infants who did not have hypothermia (n=321)** | **p-value** |
| --- | --- | --- | --- |
| Need for IV Therapy for Hypoglycemia, n (%) | 2 (9.5) | 2 (15.4) | 0.627 |
| NICU/SCN Admission, n (%) | 14 (66.7) | 7 (53.8) | 0.491 |
| Length of Stay in hours, mean (SD) | 96.0 (90.8) | 74.4 (49.5) | 0.254 |
| Need for Respiratory Support, n (%) | 5 (23.8) | 0 (0) | 0.132 |
| Transfer to Another Hospital, n (%) | 1 (4.8) | 1 (7.7) | 1 |
| Hypoglycemia ≤ 2.6 mmol/L in First 2 Hours, n (%) | 10 (47.6) | 4 (30.8) | 0.477 |
| Hypoglycemia ≤ 2.6mmol/L in First 12 Hours, n (%) | 12 (57.1) | 5 (38.5) | 0.481 |

Insufficient data for non-invasive ventilation duration in hours, metabolic acidemia, and diagnosis of RDS.

**Table 4:** Univariate Analysis of Clinical Outcomes in Term Infants

| **Clinical Outcomes** | **Infants who had hypothermia (n=118)** | **Infants who did not have hypothermia (n=321)** | **p-value** |
| --- | --- | --- | --- |
| Need for IV Therapy for Hypoglycemia, n (%) | 4 (4.1) | 1 (0.3) | 0.013* |
| NICU/SCN Admission, n (%) | 12 (12.4) | 16 (5.2) | 0.021* |
| Length of Stay in hours, mean (SD) | 41.4 (21.5) | 34.9 (18.3) | 0.091 |
| Need for Respiratory Support, n (%) | 6 (6.2) | 7 (2.3) | 0.09 |
| Transfer to Another Hospital, n (%) | 4 (4.1) | 6 (1.9) | 0.26 |
| Non-Invasive Ventilation Duration in hours, mean (SD) | 14.1 (17.3) | 8.1 (6.5) | 0.758 |
| Metabolic Acidemia, n (%) | 1 (1.0) | 4 (1.3) | 1 |
| Diagnosis of RDS, n (%) | 0 (0) | 1 (0.3) | 1 |
| Hypoglycemia ≤ 2.6 mmol/L in First 2 Hours, n (%) | 4 (4.1) | 5 (1.6) | 0.227 |
| Hypoglycemia ≤ 2.6mmol/L in First 12 Hours, n (%) | 6 (6.2) | 14 (4.5) | 0.59 |

Note: *p-value ≤ 0.05
